# Proper modulation of AHR signaling is necessary for establishing neural connectivity and oligodendrocyte precursor cell development in the embryonic zebrafish brain

**DOI:** 10.3389/fnmol.2022.1032302

**Published:** 2022-11-29

**Authors:** Nathan R. Martin, Ratna Patel, Michelle E. Kossack, Lucy Tian, Manuel A. Camarillo, Layra G. Cintrón-Rivera, Joseph C. Gawdzik, Monica S. Yue, Favour O. Nwagugo, Loes M. H. Elemans, Jessica S. Plavicki

**Affiliations:** ^1^Department of Pathology and Laboratory Medicine, Brown University, Providence, RI, United States; ^2^Molecular and Environmental Toxicology Center, University of Wisconsin at Madison, Madison, WI, United States; ^3^Division of Pharmaceutical Sciences, University of Wisconsin at Madison, Madison, WI, United States; ^4^Department of Biology, University of Maryland Baltimore County, Baltimore, MD, United States; ^5^Division of Toxicology, Institute for Risk Assessment Sciences (IRAS), Utrecht University, Utrecht, Netherlands

**Keywords:** TCDD, dioxin, aryl hydrocarbon receptor, ahr2, neurotoxicity, olig2, zebrafish

## Abstract

2,3,7,8-tetrachlorodibenzo-[p]-dioxin (TCDD) is a persistent global pollutant that exhibits a high affinity for the aryl hydrocarbon receptor (AHR), a ligand activated transcription factor. Epidemiological studies have associated AHR agonist exposure with multiple human neuropathologies. Consistent with the human data, research studies using laboratory models have linked pollutant-induced AHR activation to disruptions in learning and memory as well as motor impairments. Our understanding of endogenous AHR functions in brain development is limited and, correspondingly, scientists are still determining which cell types and brain regions are sensitive to AHR modulation. To identify novel phenotypes resulting from pollutant-induced AHR activation and *ahr2* loss of function, we utilized the optically transparent zebrafish model. Early embryonic TCDD exposure impaired embryonic brain morphogenesis, resulted in ventriculomegaly, and disrupted neural connectivity in the optic tectum, habenula, cerebellum, and olfactory bulb. Altered neural network formation was accompanied by reduced expression of synaptic vesicle 2. Loss of *ahr2* function also impaired nascent network development, but did not affect gross brain or ventricular morphology. To determine whether neural AHR activation was sufficient to disrupt connectivity, we used the Gal4/UAS system to express a constitutively active AHR specifically in differentiated neurons and observed disruptions only in the cerebellum; thus, suggesting that the phenotypes resulting from global AHR activation likely involve multiple cell types. Consistent with this hypothesis, we found that TCDD exposure reduced the number of oligodendrocyte precursor cells and their derivatives. Together, our findings indicate that proper modulation of AHR signaling is necessary for the growth and maturation of the embryonic zebrafish brain.

## Introduction

Brain development is a complex process requiring the orchestrated differentiation of functionally distinct, but interdependent cell types. Early neural networks are established during development and subsequently modified in response to stimuli, a crucial refinement process by which information is extracted from the world and retained as memory. Despite the inherent plasticity of the brain, its development can be disrupted by a number of factors including nutritional deficiencies, infections, injury, and exposure to environmental contaminants.

2,3,7,8-tetrachlorodibenzo-[p]-dioxin (dioxin, TCDD) is a persistent global pollutant that exhibits a high affinity for the aryl hydrocarbon receptor (AHR), a ligand activated transcription factor that is broadly expressed and highly conserved in vertebrate species (reviewed in (Nguyen and Bradfield, 2008). The AHR was first identified due to its role in cellular detoxification and was subsequently found to have endogenous functions in development (Lahvis et al., 2000; Huang et al., 2004; Kim et al., 2006; Stevens et al., 2009; Mulero-Navarro and Fernandez-Salguero, 2016). A variety of compounds can activate AHR including phytochemicals found in our diets, microbial metabolites generated in the gut, and physiological ligands such as tryptophan and indole metabolites (Quintana, 2013; Quintana and Sherr, 2013; Gutiérrez-Vázquez and Quintana, 2018). Loss of AHR function disrupts the development of the central and peripheral nervous systems (CNS and PNS) in both invertebrate and vertebrate model organisms, leading to functional deficits. Impaired development and altered behavior are due, in part, to altered connectivity in the CNS and PNS. Loss of the invertebrate homologs of AHR, *spineless* (*D. melanogaster*) and *AHR-1* (*C. elegans*) disrupts specification of neuronal cell types, axon pathfinding, and dendritic branching and, in mammals, loss of *Ahr* disrupts hippocampal neurogenesis, granule neuron morphology, synaptic maturation in the adult hippocampus, and hippocampus-dependent memory (Huang et al., 2004; Qin and Powell-Coffman, 2004; Crews and Brenman, 2006; Kim et al., 2006; Latchney et al., 2013; Smith et al., 2013; Hahn et al., 2017). *Ahr* deficiency also disrupts myelin sheath formation in the optic nerve leading to congenital nystagmus in mice, a clinical feature maintained in humans with *AHR* mutations (Juricek et al., 2017; Mayer et al., 2019). Furthermore, loss of *ahr2*, a zebrafish homolog of *AHR*, produces hyperactive larval behavior and altered startle and predator avoidance responses in adults (Garcia et al., 2018).

In addition to its activation by endogenous ligands, the AHR is also stimulated by exogenous environmental ligands generated by natural events such as forest fires and volcanic eruptions as well as anthropogenic activities such as fuel and waste combustion, smoking, and traffic related air pollution (Liem et al., 2000). A large number of exogenous AHR ligands are present in our environment as chemical contaminants, including chlorinated dibenzo-p-dioxins, polychlorinated dibenzofurans, polycyclic aromatic hydrocarbons, and dioxin-like polychlorinated biphenyls (reviewed in White and Birnbaum (2009)). These global contaminants are usually found as mixtures and are highly stable and lipophilic, leading to their bioaccumulation and biomagnification in the food chain. A significant portion of what we know about the effects of AHR agonist exposure on human health comes from studies of widescale human exposure events that have occurred as result of war, industrial accidents, and food contamination. For instance, during the Vietnam War, the U.S. Air Force used Agent Orange, a defoliant contaminated with TCDD, to reduce forest coverage and, in doing so, exposed enlisted soldiers and Vietnamese civilians to high levels of TCDD. Exposures led to increased incidence of thyroid disease, diabetes, peripheral neuropathy, ischemic heart disease, and retinopathy in Vietnam veterans (Kim et al., 2003; Pavuk et al., 2003). In Vietnamese children, prenatal exposures to Agent Orange has been associated with poor social–emotional skills (Pham et al., 2015), autistic traits (Nishijo et al., 2014), and impaired motor coordination skills (Tran et al., 2016). Developmental AHR agonist exposures in other human populations have been associated with neurobehavioral conditions such as impaired hearing, altered psychomotor function, and ADHD (Perera et al., 2012, 2014; Peterson et al., 2015; Lenters et al., 2019; reviewed in White and Birnbaum, 2009). Together, these studies highlight the broad impacts of TCDD exposure on human health and suggest that TCDD targets multiple organ systems, including the nervous system.

Epidemiological studies examining the adverse effects of contaminant-induced AHR activation on brain health have been corroborated by laboratory investigations using animal models to understand the impact of AHR agonist exposure on brain development and behavior. Studies in multiple model organisms have demonstrated that developmental exposure to AHR agonists produces functional changes in the nervous system (Dubovický et al., 2008; Chen et al., 2012; Knecht et al., 2017; Gileadi et al., 2021; Holloway et al., 2021). In rats, exposure to the AHR agonist benzo[a]pyrene (BaP) during critical postnatal periods of development resulted in impaired learning and memory with deficiencies being persistent and exacerbated with age (Chen et al., 2012). TCDD exposure in adult mice impairs neurogenesis and neuronal differentiation in the hippocampus as well as hippocampal-dependent contextual memory (Latchney et al., 2013). Low-dose, gestational TCDD exposure in mice was found to induce motor and learning deficits in juvenile females (Gileadi et al., 2021) and, in zebrafish, embryonic BaP exposure has been reported to produce larval hyperactivity as well impaired learning in adult fish (Knecht et al., 2017). However, under different exposure paradigms and testing parameters, BaP exposure was also reported to produce hypoactivity in zebrafish (Holloway et al., 2021).

The described studies have made important contributions to our understanding of endogenous AHR functions in nervous system development as well as how exposure to AHR agonists can adversely affect brain health. However, prior studies have only focused on a subset of brain regions and cell-types. Ahr2 along with its cofactors are expressed in the developing zebrafish CNS and previous work has shown AHR agonist exposure results in the induction of biomarkers of AHR activation throughout embryogenesis and early larval development (Andreasen et al., 2002; Yin et al., 2008; Jenny et al., 2009; Kubota et al., 2011). To gain additional insight into the brain regions and cell-types that are affected by AHR activation and loss of function, we exposed zebrafish to TCDD during early embryogenesis. Zebrafish develop externally and are transparent allowing researchers to visualize embryonic brain development *in vivo* and *ex utero*; studies that would be challenging or not possible in other vertebrate model systems. We found that early embryonic TCDD exposure disrupted development of the habenula, the pallium, olfactory bulb, cerebellum, and optic tectum. Exposure also disrupted ventricular morphogenesis, impaired neural network formation, and reduced the number of oligodendrocyte precursor cells and their derivatives. To assess the sensitivity of these endpoints to *ahr2* loss of function, we examined a subset of endpoints in *ahr2* mutant embryos. Loss of *ahr2* disrupted neural network formation but did not affect gross brain or ventricular morphology. Embryos and larvae heterozygous for *ahr2* were phenotypically similar to *ahr2* null mutant, indicating that *ahr2* haploinsufficiency disrupts neural network formation. Our findings are consistent with previous mammalian reports and also identify new brain regions sensitive to AHR modulation.

## Materials and methods

### Zebrafish husbandry

All colony maintenance, procedures, and experimental techniques pertaining to zebrafish were approved by the Brown University Institutional Animal Care and Use Committee (IACUC) and adhere to the National Institute of Health’s “Guide for the Care and Use of Laboratory Animals.” Transgenic zebrafish lines were maintained in an aquatic housing system (Aquaneering Inc., San Diego, CA) with centralized filtration, reverse osmosis (RO) water purification, automatic pH and conductivity stabilization, consistent temperature maintenance (28.5 ± 2°C), 14-h: 10-h light–dark cycle, and ultraviolet (UV) irradiation for microorganism disinfection, as according to Westerfield (2000). The Plavicki Lab Zebrafish Facility undergoes routine monitoring for disease including the semiannual quantified Polymerase Chain Reaction (qPCR) panels to detect common fish pathogens.

Adult zebrafish were placed into 1.7 l sloped spawning tanks (Techniplast, USA) 15–18 h prior to breeding. Sexes were separated by a transparent partition. Within 2 h of light cycle onset, the partition was removed, and zebrafish were allowed to spawn for 1 h. Embryos were collected in fresh egg water (60 mg/l Instant Ocean Sea Salts; Aquarium Systems, Mentor, OH) and placed into 100 mm non-treated culture petri dishes (CytoOne, Cat. No. CC7672-3394) until time of toxicant exposure. Embryonic and larval zebrafish were maintained at 28.5 ± 1°C in an incubator (Powers Scientific Inc., Pipersville, PA) up to 72 h post fertilization (hpf). Previously established transgenic lines were used to examine the effects of TCDD exposure on brain morphology and neuronal and glial development (Table 1).

**Table 1:** Transgenic *Danio Rerio* lines used in manuscript.

Transgenic Line	Tissue/Function	Reference
*Tg(olig2:EGFP)^vu12^*	OPCs and motor neurons	Shin et al. (2003)
*Tg(sox10:RFP)*	Neural crest and differentiated oligodendrocytes	Kucenas et al. (2008)
*Tg(elavl3:Gal4-VP16)*	Elavl3 promoter fused Gal4-driver enabling neuronal specific expression of genes fused to an upstream-activating sequence (UAS)	Kimura et al. (2008)
*Tg(UAS:caAHR:2A-tRFP:cryaa:EGFP)*	Paired with Gal4-driver, enables expression of constitutively active AHR in cell-specific manner	Current article
*ahr2^uab147^*	Frameshift mutation in AHR2, resulting in loss-of-function and sensitivity to TCDD toxicity	Souder and Gorelick (2019)

### 2,3,7,8-tetrachlorodibenzo-[p]-dioxin (TCCD, dioxin) stock preparation and exposure

TCDD exposures were performed as described by Plavicki et al. (2013). TCDD (ED-901-B, Cerriliant, St. Louis, MO) stock solutions were prepared by diluting 50.00 ± 0.32 μg/ml TCDD with pure dimethyl sulfoxide (DMSO) to a final concentration of 9.973 ± 0.223 μg/ml as reported in Kossack et al. (2023). Stock solutions were prepared in 2 ml amber vials and caps were wrapped with Parafilm® to limit evaporation. Stock solutions were stored in the dark at room temperature.

Briefly, collected zebrafish embryos were inspected for fertilization and quality at 3 hpf. At 4 hpf, 20 healthy embryos were transferred into 2 ml amber vials (Cat. No. 5182–0558, Agilent, Santa Clara, CA) and 2 ml of egg water was added to each vial. Two micro liter of prepared TCDD stock (9.973 ± 0.223 μg/ml) was added to an amber vial containing embryos and 2 ml of egg water.

For control samples, 2 μl of DMSO (control) was added as a vehicle control exposure (0.1% DMSO). Vials were capped, wrapped in Parafilm®, and inverted several times to distribute chemical treatment. Embryos were then continuously mixed for 1 h at room temperature on a rocker table. Treatment solution was removed from the vials and embryos were washed three times with egg water. All embryos were transferred to 6-well plates (20 embryos/well) containing egg water and placed into the incubator. This established dosing paradigm is sufficient to induce prolonged AHR activation throughout embryogenesis and larval development.

At 24 hpf, embryos were manually dechorionated using Dumont #5XL forceps (Cat. No. 11253–10, Fine Science Tools, Foster City, CA). Chorion debris and egg water was removed from each well and replaced with 0.003% 1-phenyl-2-thiourea (PTU, Sigma) solution in egg water to inhibit pigment development in embryos and facilitate confocal microscopy.

Previous reports have identified phenotypic indicators of TCDD toxicity in embryos exposed to concentrations ranging from 1 ng/ml (Plavicki et al., 2013) to 10 ng/ml (Souder and Gorelick, 2019). Variability in exposure concentrations sufficient to induce TCDD toxicity across research institutions is attributable to the limited solubility of TCDD and adherence to glass surfaces. Additionally, lack of consistent stock and treatment solution validation could account for phenotypic variations following TCDD exposures. As such, TCDD stock solutions were validated as according to Kossack et al. (2023).

### Immunohistochemistry

Following TCDD or DMSO exposure, larval zebrafish were fixed in 4% paraformaldehyde (PFA, Sigma-Aldrich, Cat. No. P6148) for 18–24 h at 4°C. Post-fixation, larvae were washed 3–5 times in PBS-T (phosphate buffered solution +0.6% Triton-X 100). Embryos were bleached (MilliQ water +3% H2O2 + 1% KOH) for 20–30 min at room temperature and washed 3–5 times with PBS-T. Larvae were placed in blocking solution (PBS-T + 4% bovine serum albumin, BSA) for 18–24 h at 4°C. Samples were immunolabeled with 1^o^ antibodies for 1–2 days at 4°C. Embryos were washed 3–5 times with PBS-T to remove residual 1^o^ antibodies and placed at 4°C for 18–24 h. PBS-T solution was removed and 2^o^ antibodies were added to the embryos. The samples were immunolabeled with 2^o^ antibodies for 18–24 h at 4°C. 2^o^ antibodies were removed by washing samples 3–5 times with PBS-T. During final PBS-T wash, Hoechst stain was added (1,10,000 in PBS-T) and samples were placed at 4°C for 18–24 h. PBS-T was removed and VECTASHIELD Mounting Media (Vector Labs, Cat. No. H-1000) was added to embryos. Samples incubated for 5 min at room temperature, then were gently mixed and placed in -20°C until imaging. The following primary antibodies were used: acetylated-α-tubulin (1:250, mouse monoclonal [IgG2b], Sigma-Aldrich, Cat. No. T7451) and synaptic vesicles 2 (SV2 (1:100, mouse monoclonal [IgG1], Developmental Studies Hybridoma Bank). The SV2 monoclonal antibody was deposited by K.M. Buckley and obtained from the Developmental Studies Hybridoma Bank, created by the NICHD of the NIH and maintained at The University of Iowa, Department of Biology, Iowa City, IA 52242. Secondary antibodies used: Alexa Fluor 488 goat anti-mouse IgG1 (1,100, Life Technologies) or Alexa Fluor 633 goat anti-mouse IgG (H + L, 1:100, Life Technologies).

### Confocal imaging

Stained embryos were fixed in 4% paraformaldehyde for 18–24 h at 4°C. After fixation, zebrafish embryos were either immunolabeled or washed with PBS-T and placed into VECTASHIELD. Fixed embryos were removed from VECTASHIELD and mounted in 2% low-melting agarose (Fisher Scientific, bp1360-100) in a 35 mm glass bottom microwell dish (MatTek, Part No. P35G-1.5-14-C). Confocal z-stacks were acquired on a Zeiss LSM 880 confocal microscope and maximum intensity projections were generated in Zen Black (Zeiss). Z-step sizes were optimized to the objective lens being used to collect images.

### Brain morphometric analyses

Analysis of zebrafish neuroanatomical images was conducted using the open-source FIJI/ImageJ software. Maximum intensity projection images of Hoechst-stained zebrafish brains (48–52 hpf) were imported into ImageJ. Regions of interest (ROI), which included the whole brain (WB), forebrain (FB), optic tectum (OT), cerebellum (Ce), and hindbrain (HB), were manually traced using a polygon selection tool. Area was measured for all ROI, and selected ROI masks were saved for future analyses. Prior to additional measurements, each image was masked based upon whole brain ROI. Intensity-based thresholding (Image ➔ Type ➔ 8-bit ➔ Adjust ➔ Auto Threshold ➔Method ➔ Moments ➔ Ignore White ➔ Ok) was applied to images of immunolabeled samples (acetylated α-tubulin, SV2). Differences between control and treatment groups were assessed by measuring area fraction (percentage area coverage of immunolabel in each brain region). Data for control and TCDD-exposed embryos were compared to the control group average. Significant differences between groups were determined using Welch’s t-test in GraphPad Prism.

### Analysis of axon tract formation

To examine the effects of neuron-specific AHR activation, expression of a UAS-fused constitutively active AHR (caAHR) was driven by a differentiated neuron-fused Gal4 driver, *Tg(elavl3:Gal4; UAS:caAHR:2A-tRFP:cryaa:EGFP)*. Level of expression was determined by the presence of red-labeled neurons in the developing brain at 48–52 hpf. Samples were immunolabeled with acetylated α-tubulin antibodies and the percentage area coverage of immunolabel in each brain region was quantified as described above. To examine the effects of global *ahr2* loss on brain development, *ahr2^uab147^* heterozygote (*ahr2^+/−^*) zebrafish were crossed with homozygous *ahr2^uab147^* mutants (*ahr2^−/−^*) to generate a 50:50 mix of heterozygotes (*ahr2^+/−^*) and homozygous *ahr2* mutants (*ahr2^−/−^*). Samples were immunolabeled with acetylated-α-tubulin antibodies and brain morphometric analyses were performed using age matched controls. Samples were genotyped post analysis.

### Quantification of developing oligodendrocytes

The number and distribution of oligodendrocyte progenitor cells (*Tg(olig2:EGFP)^vu12^; Tg(sox10:RFP)*) in the developing zebrafish hindbrain was determined using confocal images of control and TCDD-exposed embryos at 48–50 hpf. The hindbrain was defined as the area spanning from the Purkinje cell layer of the cerebellum (Hsieh et al., 2014) to the base of the spinal cord. Positive cells were counted in two areas: along the midline and the lateral surface. Significant differences between groups were determined by Welch’s t-test using GraphPad Prism.

### Quantitative RT-PCR

Zebrafish larvae were collected at 3 dpf, pooled in groups of ten and flash frozen in liquid nitrogen. RNA isolation and purification was carried out using the RNeasy Plus Kit (QIAGEN). cDNA synthesis was achieved using the SuperScript IV Reverse Transcriptase First-Strand Synthesis System kit (Invitrogen, Cat. No. 18091050). qRT-PCR for *cyp1a1* was performed with 7.5 ng/uL of cDNA using the ViiA7 Real Time PCR System (Applied Biosystems). *cyp1a1* was detected by using a TaqMan probe (Thermo Fisher Scientific; ID: Dr03112441_m1).

## Results

### Developmental TCDD exposure impairs embryonic brain morphogenesis

**Figure 1:**
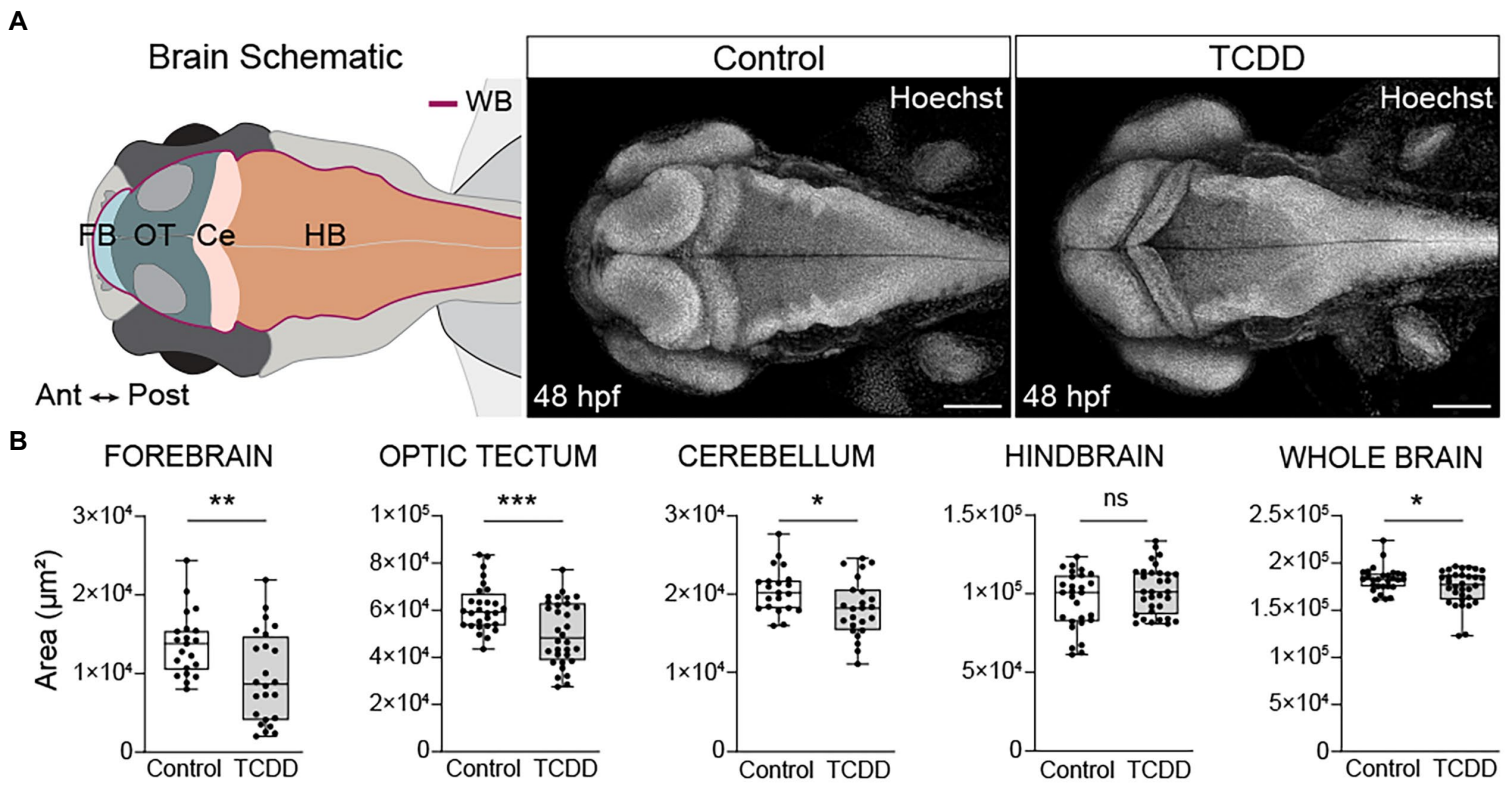
Developmental TCDD exposure impairs embryonic brain morphogenesis. **(A)** Schematic of the embryonic zebrafish brain at 48 hpf depicting the brain regions quantified in B. Dorsal confocal images of Hoechst (DNA, white) stained embryos and larvae were used to visualize and quantify differences in gross brain morphology. Embryos were exposed at 4 hpf to either control (DMSO) or TCDD (10 ppb) for 1 h. **(B)** Regional and whole brain size was determined by measuring the area of the forebrain (FB), optic tectum (OT), cerebellum (Ce), hindbrain (HB), and whole brain (WB) in control and TCDD-exposed embryos. Statistical significance determined by Welch’s t-test, *p < 0.05, **p < 0.01, ***p < 0.001. n = 21–26 fish/group across 3 replicates. Anterior (Ant) is left, posterior (Post) is right in all confocal micrographs. Scale bar = 100 μm, magnification = 10x.

**Figure 2:**
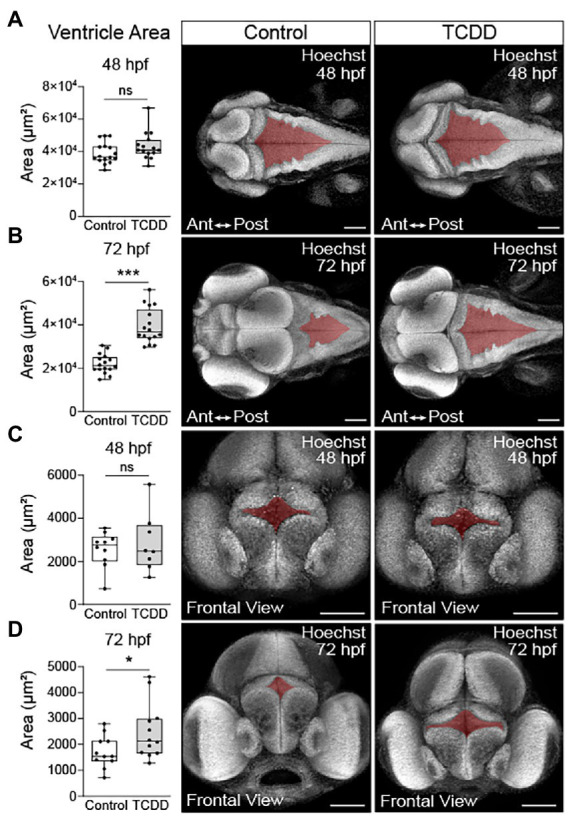
TCDD exposure increases brain ventricle size. To determine the impact of TCDD exposure on ventricular development, the hindbrain **(A,B)** and forebrain **(C,D)** ventricles were measured in embryos ranging in age from 48 to 52 hpf (A&C) and larvae from 72 to 76 hpf **(B,D)**. TCDD exposure significantly increased hindbrain and forebrain ventricle size at 72 hpf **(B,D)** with non-significant trends observed early in development. Dorsal 10x magnification **(A,B)** and frontal **(C,D)** 20x confocal micrographs of zebrafish embryos and larvae stained with Hoechst. *N* = 15–17 fish/group hindbrain across 3 replicates; *n* = 8–12 fish/group forebrain across replicates. Anterior (Ant) is left, posterior (Post) is right in confocal micrographs **(A,B)**. Scale bar = 100 μm. Statistical significance determined by Welch’s *t*-test, *p < 0.05, ****p < 0.0001.

To determine how early embryonic TCDD exposure affects brain development, we used an exposure paradigm that is sufficient to induce prolonged AHR activation throughout embryogenesis and into early larval stages [Supplementary Figure 1 and (Yue et al., 2021)]. Newly fertilized embryos were exposed to TCDD for 1 h at 4 hpf. Due to its lipophilic nature, TCDD is readily absorbed during the exposure window and remains detectable as the parent compound at 72 hpf (Kossack et al., 2023). We collected and analyzed samples at 48 hpf, a time point at which Ahr2 is still broadly expressed in the brain and biomarkers of AHR induction, including *cyp1a1*, *cyp1b1*, and *cyp1c1*, are detected in CNS following TCDD exposure ((Andreasen et al., 2002; Yin et al., 2008; Jenny et al., 2009; Kubota et al., 2011). Importantly, at 48 hpf, TCDD exposed embryos do not exhibit confounding phenotypic changes such as gross morphological malformations, impaired cardiac output, or increased mortality, all of which are factors that can hinder our ability to distinguish the effects of exogenous AHR activation on the developing nervous system from systemic effects of toxicity [Supplementary Figure 1 and (Henry et al., 1997; Dong et al., 2002; Bello et al., 2004; Antkiewicz et al., 2005; Mehta et al., 2008; Xiong et al., 2008; Teraoka et al., 2010; Plavicki et al., 2013; Burns et al., 2015)]. TCDD exposure significantly reduced the area of the forebrain, optic tectum, cerebellum, and whole brain relative to vehicle controls, but did not affect hindbrain size at 48 hpf **(** Figure 1 **)**. To determine if the observed changes in brain size were the result of a developmental delay, we collected an additional set of samples at 72 hpf and found the observed reductions in the size of forebrain, optic tectum, and cerebellum persisted in the TCDD exposed group **(** Supplementary Figure 2 **)**. In contrast to the phenotype observed at 48 hpf, the hindbrain was significantly larger at 72 hpf in TCDD-exposed zebrafish and, as a result of hindbrain enlargement, there was no difference in whole brain size between treatment groups at 72 hpf **(** Supplementary Figure 2C). To examine whether the increase in hindbrain size was the result of ventriculomegaly, the enlargement of the brain ventricles, we measured ventricular size in the forebrain and hindbrain at 48 and 72 hpf. TCDD exposure did not significantly alter ventricle size at 48 hpf (Figures 2A,C); however, both the hindbrain and forebrain ventricle areas in TCDD-exposed larvae were significantly enlarged at 72 hpf (Figures 2B,D).

### Embryonic TCDD exposure disrupts neural network formation

**Figure 3:**
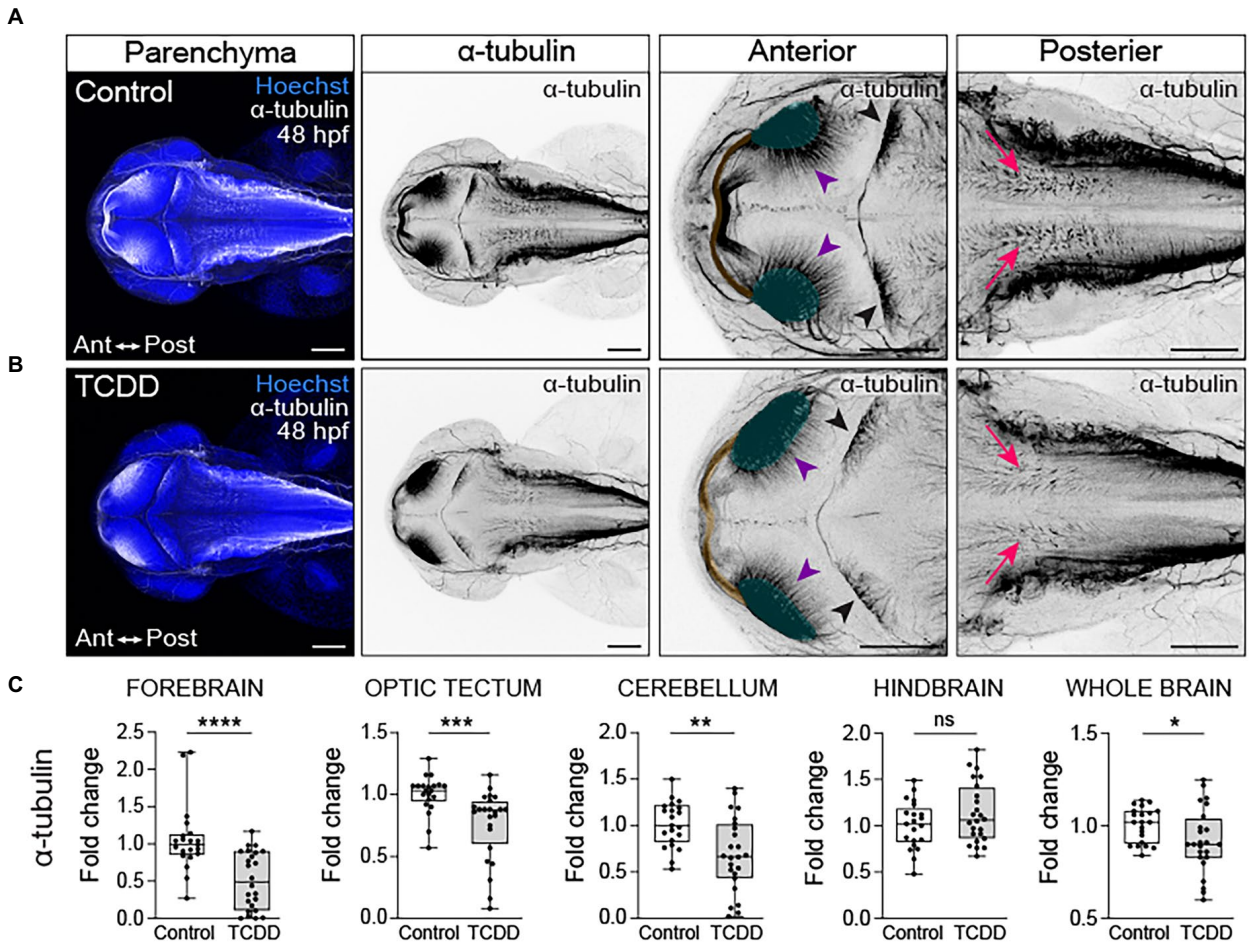
Embryonic TCDD exposure disrupts neural network formation. Dorsal confocal images of axon tracts in control **(A)** and TCDD-exposed **(B)** embryos at 48 hpf. The brain parenchyma is marked by Hoechst staining (DNA, blue) and axon tracts are immunolabeled with an anti-acetylated α tubulin (α-tubulin) antibody (white and inverted black signal). Projections emanating from the optic neuropils (green shading and purple arrowheads) were reduced in TCDD-exposed embryos. The posterior commissure (yellow shading) appears defasciculated following TCDD exposure. Purkinje cells in the cerebellum (black arrowheads) were malformed in TCDD-exposed zebrafish relative to controls. Although the area covered by acetylated α-tubulin in the hindbrain was not statistically different between groups, we noted that the acetylated α-tubulin projections in the hindbrain (pink arrows) appear diminished in TCDD-exposed embryos. **(C)** Fold change of acetylated α-tubulin in the forebrain, optic tectum, cerebellum, hindbrain, and whole brain calculated relative to average α-tubulin area coverage in controls. Statistical significance determined by Welch’s *t*-test, *p < 0.05, **p < 0.01, ***p < 0.001, ****p < 0.0001. *n* = 21–26 fish/group across 3 replicates. Anterior (Ant) is left in all confocal micrographs. First 2 panels containing confocal micrographs in A and B are at a 10x magnification. All other confocal micrographs are at a 10x magnification with a 2.5 digital zoom). Scale bar = 100 μm.

**Figure 4:**
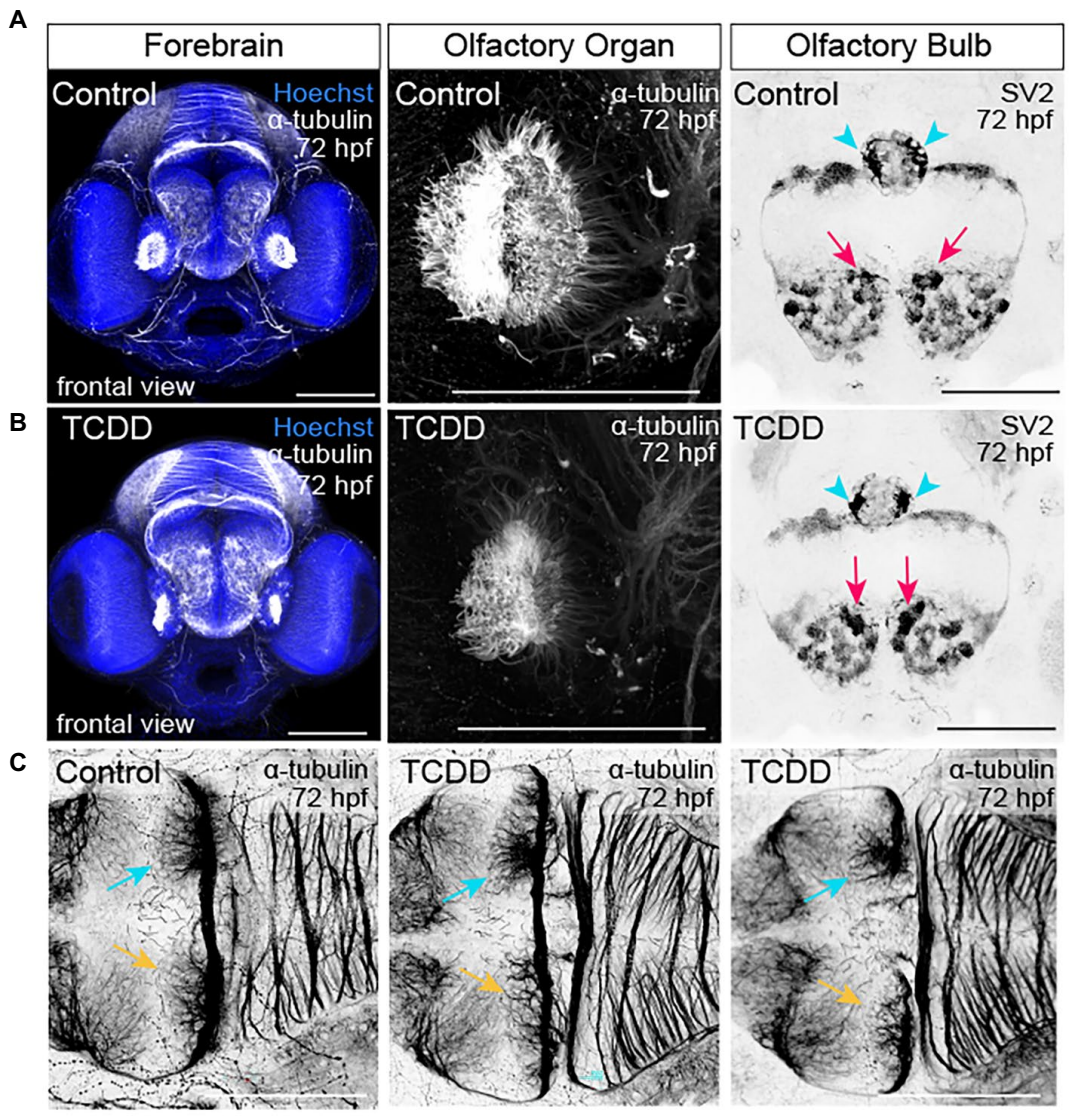
TCDD exposure disrupts development of the olfactory organs, olfactory bulbs, and habenular nuclei. **(A,B)** Frontal views of the embryonic forebrain at 72 hpf in control **(A)** and TCDD-exposed **(B)** larval zebrafish. Embryos were immunolabeled with an anti-acetylated α-tubulin antibody to visualize developing axon tracks (white and inverted to black) and stained with Hoechst (DNA, blue) to mark the parenchyma. Glomeruli in the olfactory bulb were visualized using anti-synaptic vesicles 2. TCDD exposure disrupted development of both the olfactory organs and the glomeruli in the olfactory bulbs. Pink arrows indicate the medial glomerular clusters in the olfactory organs. Mitral cells in medial glomerular clusters make asymmetric, direct projections to the right habenular nuclei (aqua arrows, yellow arrows indicate the left nuclei). **(C)** Development of the habenular nuclei was impaired at 48 hpf (see Supplementary Figure 4) and abnormal in all larvae scored at 72 hpf. Two examples of the habenular phenotypes are shown in C. Olfactory organ: *n* = 10 fish/group across 3 replicates. Olfactory bulb: *n* = 7–10 fish/group across 2 replicates. Habenula: *n* = 7–10 fish/group across 2 replicates. The dorsal surface of the brain is at the top and the ventral surface is at the bottom in **(A,B)**. Anterior is at the top in **(C)**. Scale bar = 100 μm.

To examine how TCDD exposure impacts the development of nascent axon tracts, we used the previously described dosing paradigm and performed fluorescent immunohistochemistry using an anti-acetylated α-tubulin antibody to label axon tracts in embryos and larvae. We subsequently analyzed confocal micrographs to assess the development of neuronal tracts within different brain regions. TCDD treatment grossly decreased neural network formation at 48 hpf **(** Figure 3). Significant reductions in axonal projections were observed across multiple brain regions, including the forebrain, optic tectum, cerebellum, and whole brain **(** Figure 3). Within the forebrain, development of the olfactory bulb was disrupted by TCDD exposure (Figure 4). Eighty-five percent of the larvae in the TCDD exposure group had olfactory bulbs in which the glomeruli were reduced in number and had less discrete structures (pink arrows in Figure 4). The altered glomerular structure was associated with and likely resulted at least in part from altered development of the olfactory organs. Development of the olfactory organs was disrupted in 100% of TCDD-exposed larvae scored. We also observed reduced axonal projections innervating of the habenula at 48 hpf (Supplementary Figure 3) and aberrant innervation of the habenula at 72 hpf in 100% of the embryos imaged (pink arrowheads in Figure 4). Given that mitral cells within the olfactory bulb send projection to the habenula, the observed abnormal development of the olfactory bulbs could also contribute to the habenular phenotypes. In addition, we observed, but did not quantify aberrant patterns of fasciculation at the forebrain-midbrain boundary, an increase in axonal tracts extending towards the midline in the anterior portion of the optic tectum (yellow arrowheads in Supplementary Figure 3), and abnormal development of the pineal gland (aqua arrowheads in Supplementary Figure 3).

In the optic tectum, TCDD exposure impaired development of the posterior commissure and optic tectum neuropils (Figures 3A,B). Nerve bundles in the posterior commissure appear defasciculated (Figure 3B) in TCDD-exposed zebrafish and there was a marked reduction of axon tracts projecting from the neuropils into the optic tectum at 48 hpf (purple arrowheads in Figures 3A,B). This phenotype was also present in 72 hpf TCDD treated larvae with apparent reductions in the intertectal fascicles and commissures crossing the midline (purple arrowheads in Supplementary Figures 2A,B). Furthermore, TCDD exposure altered the morphology of Purkinje cells [pink arrows in Supplementary Figure 4, (Hsieh et al., 2014)]. The Purkinje cells in the control embryos creating a fan like appearance that occupied a greater area of the cerebellum in a uniform pattern, whereas the Purkinje cells in the TCDD exposed embryos and larvae were irregularly clumped, potentially reflecting aberrant pattens of fasciculation. These phenotypes are consistent with the observed reduction in acetylated alpha tubulin coverage in the cerebellum reported in Figure 2.

In the hindbrain, there were no differences in the overall area occupied by acetylated α-tubulin in TCDD-exposed embryos and larvae when compared to controls; however, we noted that within the rhombomeres that acetylated α-tubulin+ structures were less defined. In the control hindbrain, nerve bundles were dense and created clearly defined structures oriented in a dorsal ventral manner. Following TCDD exposure, nerve bundles were diffuse and occasionally angled in an anterior orientation **(**pink arrowheads in Figure 3 and Supplementary Figure 2).

**Figure 5:**
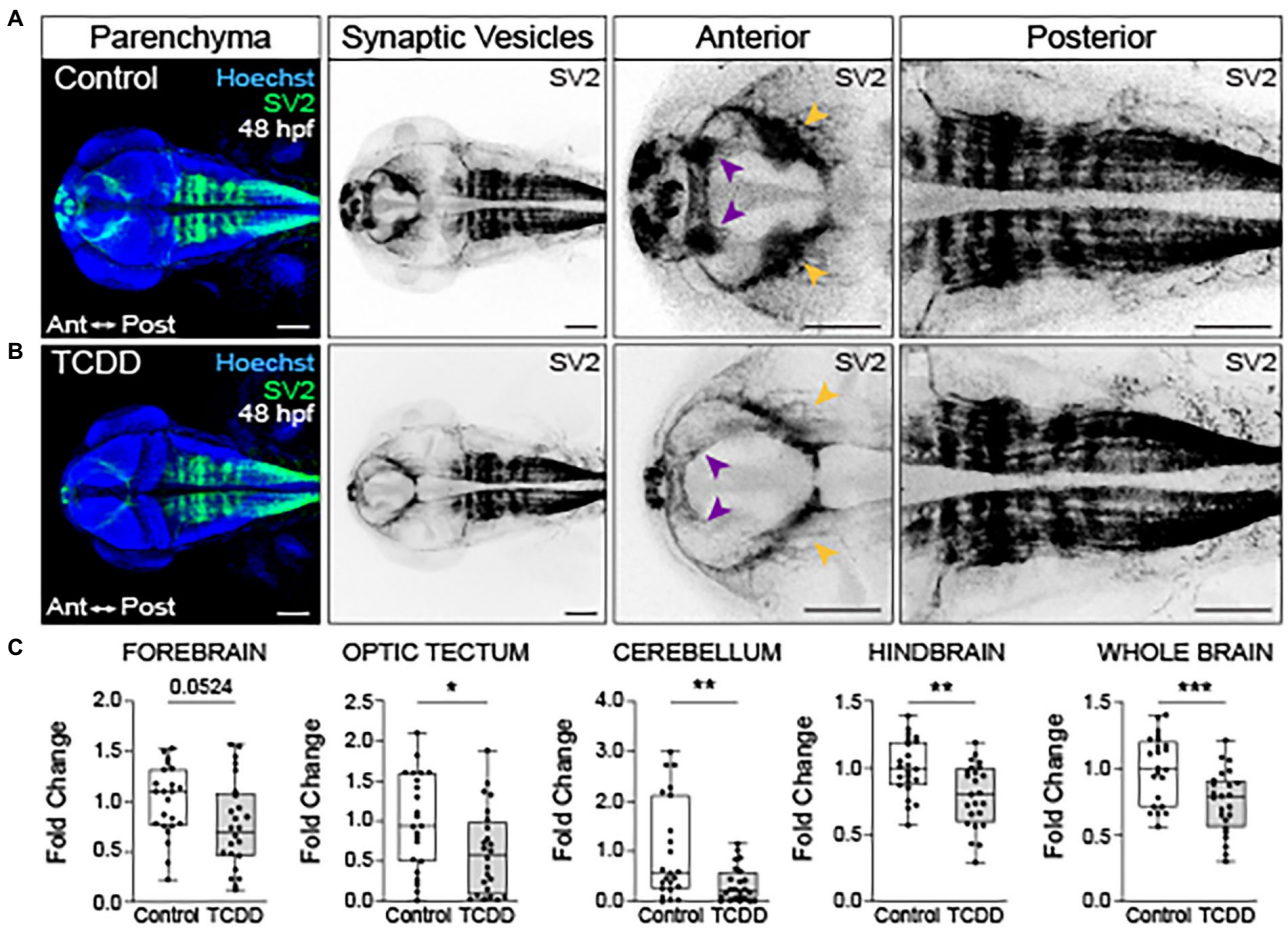
Embryonic TCDD exposure reduces synaptic vesicle 2 immunoreactivity. Dorsal confocal images of control **(A)** and TCDD-exposed **(B)** zebrafish at 48 hpf. The brain parenchyma was stained with Hoechst (DNA, blue) and immunolabeled with an antibody marking synaptic vesicle glycoprotein 2 (SV2) (green and inverted black). Forebrain vesicle production was decreased following TCDD exposure with notable reductions in the habenula (purple arrowheads). SV2 immunoreactivity was significantly reduced in the optic tectum (yellow arrowheads), cerebellum and hindbrain, and whole brain following TCDD exposure. **(C)** Fold change of SV2 immunolabeling in the forebrain, optic tectum, cerebellum, hindbrain, and whole brain was calculated relative to mean SV2 area coverage of controls. Statistical significance was determined by Welch’s *t*-test, *p < 0.05, **p < 0.01, ***p < 0.001. *n* = 23–24 fish/group across 3 replicates. Scale bar = 100 μm. Anterior (Ant) is left in all confocal micrographs.

To further examine the impact of global AHR activation on neural connectivity in the embryonic brain, we used fluorescent immunohistochemistry to detect synaptic vesicle glycoprotein 2 (SV2) expression in both TCDD-exposed and control embryos and larvae (Serajee and Huq, 2015; Taoufik et al., 2018). SV2 is a family of proteins (SV2A, SV2B, SV2C) with functions in exocytotic release of neurotransmitters and hormones, trafficking, recycling, and calcium-dependent neurotransmission (Crowder et al., 1999; Stout et al., 2019; Fronczak et al., 2021). Developmental TCDD exposure reduced SV2 immunoreactivity in the forebrain (*p* = 0.0524) with significant reductions observed in the optic tectum, cerebellum, hindbrain, and whole brain in TCDD-exposed embryos (Figure 5). Consistent with the axon tract phenotypes observed in TCDD-exposed zebrafish, SV2 immunoreactivity was reduced in the olfactory bulbs, habenula, and optic tectum neuropils following TCDD exposure (purple and yellow arrowheads in Figure 5) relative to controls. SV2 labeling clearly identified rhombomere structures in the embryonic zebrafish hindbrain (Figures 5A,B). TCDD exposure decreased SV2 labeling of interstitial spaces between rhombomeres (Figure 5).

### Early TCDD exposure reduces oligodendrocyte number

**Figure 6:**
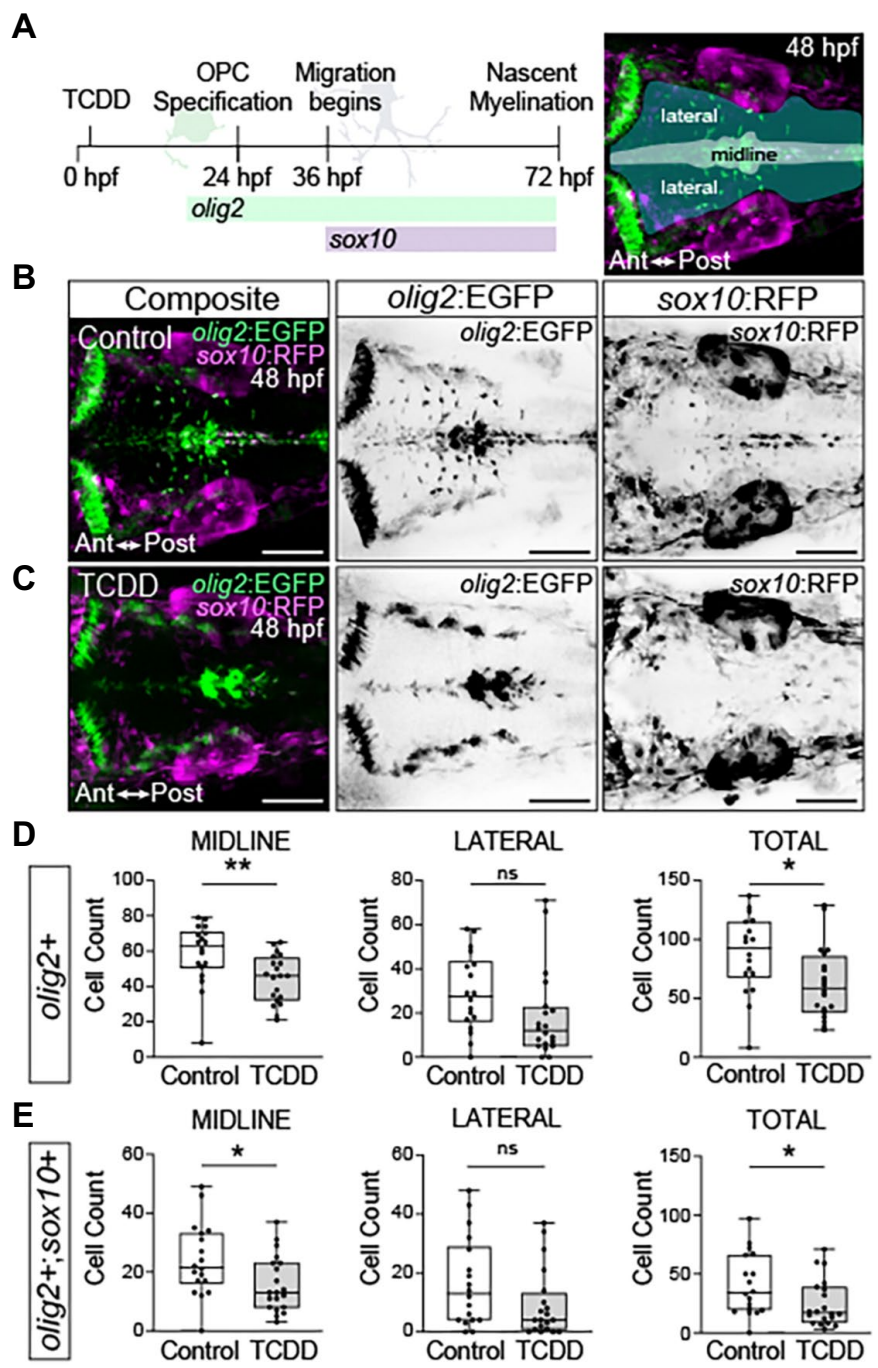
Embryonic TCDD exposure reduces oligodendrocyte numbers in the developing hindbrain. **(A)** Timeline of oligodendrocyte percursor cell (OPC) development and dorsal confocal images of the hindbrain at 48 hpf from transgenic zebrafish carrying fluorescent reporter genes for *olig2* and *sox10* [*Tg(olig2:EGFP; sox10:RFP)*]. Expression of *olig2* marks oligodendrocyte precursors, oligodendrocytes, and motor neurons. Differentiated oligodendrocytes express *sox10*, which also marks migrating neural crest cells and other neural crest derived lineages. *olig2+* cells were quantified along the midline, laterally and in total for the developing hindbrain of control **(B)** and TCDD-exposed **(C)** embryos. **(D,E)** Co-expression of *olig2* driven EGFP and *sox10* driven RFP was used to identify and quantify differentiated oligodendrocytes in the hindbrain. Cells were quantified along the midline, where OPCs are located, and in lateral domains containing OPC derived motor neurons and differentiated oligodendrocytes. Statistical significance was determined by Welch’s *t*-test. *p < 0.05, **p < 0.01. *n* = 18–20 fish/group across 3 replicates. Anterior (Ant) is left in all confocal micrographs. Scale bar = 100 μm. Magnification = 20x.

Prior studies identified oligodendrocytes as possible targets of developmental and adult TCDD toxicity in the hippocampus, cerebellum, and medulla oblongata (Fernández et al., 2010; Rosińczuk et al., 2015; Gileadi et al., 2021). These studies were limited to gene expression data from homogenized tissues and analysis of histological sectioning. To better understand the spatial and temporal effects of TCDD exposure on oligodendrocyte development, we used a transgenic line, *Tg(olig2:EGFP)^vu12^,* which marks neuroepithelial precursors that give rise to a subset of oligodendrocyte precursor cells and oligodendrocytes in the hindbrain as well as motor neurons (Shin et al., 2003; Zannino and Appel, 2009). To distinguish motor neurons from oligodendrocyte precursor cells and oligodendrocytes, we crossed *Tg(olig2:EGFP)^vu12^* to *Tg(sox10:RFP*; Zannino and Appel, 2009)*. sox10* is a SoxE transcription factor necessary for oligodendrocyte differentiation, migration, myelination, and survival (Stolt et al., 2002; Finzsch et al., 2008; Takada et al., 2010). Therefore, migrating oligodendrocyte precursor cells will appear *olig2+, sox10+* and motor neurons, which emerge from r5 and r6 rhombomeres in the ventral medial neural tube between 33–48 hpf in the developing zebrafish hindbrain, will appear *olig2+, sox10*-, (Zannino and Appel, 2009). *olig2+, sox10-* cells and *olig2+, sox10+* cells were quantified along in ventral medial region (midline) and laterally (migrating) at 48 hpf following TCDD treatment **(** Figure 6). TCDD exposure reduced *olig2+, sox10-* cells at the midline and in total (Figure 6D), which may reflect loss of neuroepithelial progenitors and/or motor neurons. TCDD exposure also reduced the number of *olig2+, sox10+* oligodendrocytes along the midline and in total within the developing zebrafish hindbrain, which reflects a loss of oligodendrocyte precursors and/or oligodendrocytes (Figure 6E).

### Neuron-specific expression of caAHR disrupts development of the cerebellum

**Figure 7:**
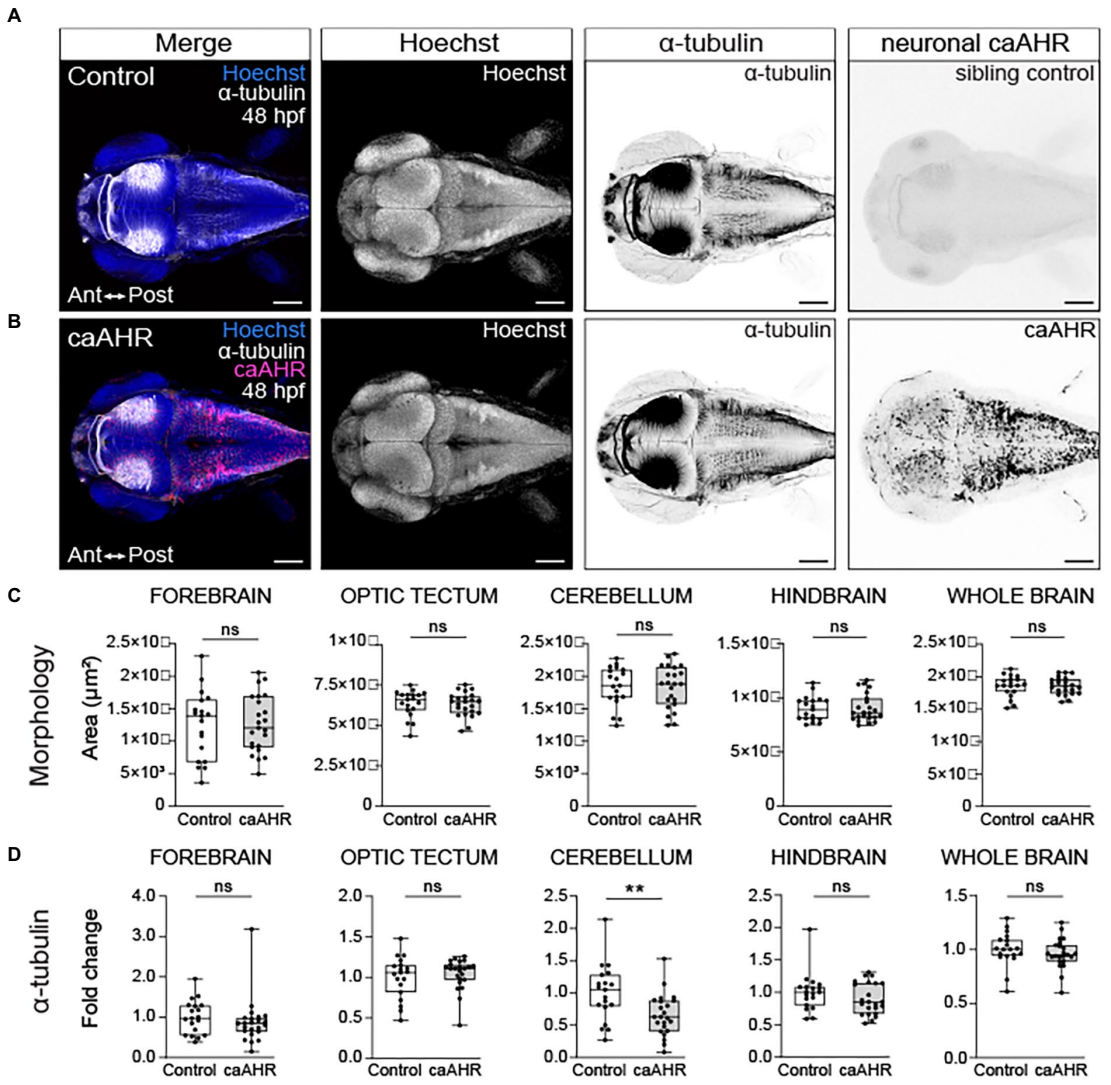
Neuron-specific activation of AHR disrupts the development of the cerebellum without affecting brain regions disrupted by global AHR activation. Confocal images at 48- hpf of control embryos **(A)** and embryos with neuron-specific expression of a constitutively active AHR [*Tg(elav3l:Gal4;UAS:caAHR:2A-tRFP)*] **(B)**. Embryos were stained with Hoechst (blue in the first panel and white in second panel of both **A,B**) to visualize the brain parenchyma and immunolabeled with an anti-acetylated alpha tubulin antibody (α-tubulin) to mark axon tracts (white in the first panel and inverted black in the third panel of both **A,B**). There were no differences in the morphology of the forebrain, optic tectum, cerebellum, hindbrain, and whole brain at 48 hpf when comparing control **(C)** embryos to embryos with neuron-specific expression of a constitutively active AHR. **(D)** Fold change in acetylated α-tubulin expression in the forebrain, optic tectum, cerebellum, hindbrain, and whole brain in control and *Tg(elav3l:Gal4;UAS:caAHR:2A-tRFP* embryos relative to mean area coverage of α-tubulin in wild-type embryos. Statistical significance determined by Welch’s *t*-test, *p < 0.05, **p < 0.01, ***p < 0.001. *n* = 19–23 fish/group across 3 replicates. Anterior (Ant) is left in all confocal micrographs. Scale bar = 100 μm. Magnification = 10x.

To determine if neuron-specific activation of AHR recapitulates the axon tract defects observed in TCDD-exposed embryos, we generated a UAS line with a 2A-tRFP tagged constitutively active *AHR* construct (caAHR; Lanham et al., 2014) and drove expression of the construct using a differentiated neuron-specific Gal4 line, *Tg(elavl3:Gal4; UAS:caAHR:2A-tRFP:cryaa:EGFP)*. We confirmed that the constitutively active *AHR* construct induced expression of *cyp1a1*, a biomarker of AHR activation, in the brain (Supplementary Figure 5). The Gal4-UAS approach resulted in mosaic *caAHR* induction in the brain at 48 hpf; therefore, only embryos with broad expression were selected for assessment (Figure 7). Axonal projections into the cerebellum were reduced in embryos with neuron-specific caAHR expression (Figure 7D); however, there were no quantifiable differences in acetylated α-tubulin immunoreactivity the forebrain, optic tectum, cerebellum, hindbrain, or whole brain (Figure 7D). Furthermore, no gross morphological differences in axon tract development were noted in other brain regions (Figure 7C). Together, these findings suggest that there are additional cellular targets in the nervous system, or that differences exist between the caAHR construct and TCDD-induced AHR activation that contribute to the observed changes in brain size and axon tract development in TCDD-exposed embryos.

### Loss of *ahr2* disrupts neural network formation without affecting brain size

Mutations in *ahr2* or components of the AHR signaling pathway can be protective for organisms living in environments contaminated by AHR agonists; however, there can be fitness costs associated with impaired AHR function (Wirgin et al., 2011; Reitzel et al., 2014; Bravo-Ferrer et al., 2019; Zhang et al., 2019) [reviewed in (Whitehead et al., 2017)]. Similarly, loss of *ahr2* protects zebrafish against AHR-induced toxicity; however, loss of *ahr2* function decreases long-term survival and can disrupt developmental processes including craniofacial and ovarian development (Plavicki et al., 2013; Knecht et al., 2017; Garcia et al., 2018; Souder and Gorelick, 2019). Loss of *AHR* homologs in invertebrates disrupts neural connectivity by causing axonal branching and migration defects (Qin and Powell-Coffman, 2004; Kim et al., 2006). Using a combination of fluorescent immunohistochemistry and confocal microscopy, we examined how *ahr2* loss impacted brain morphology and axon tract development in embryonic zebrafish.

**Figure 8:**
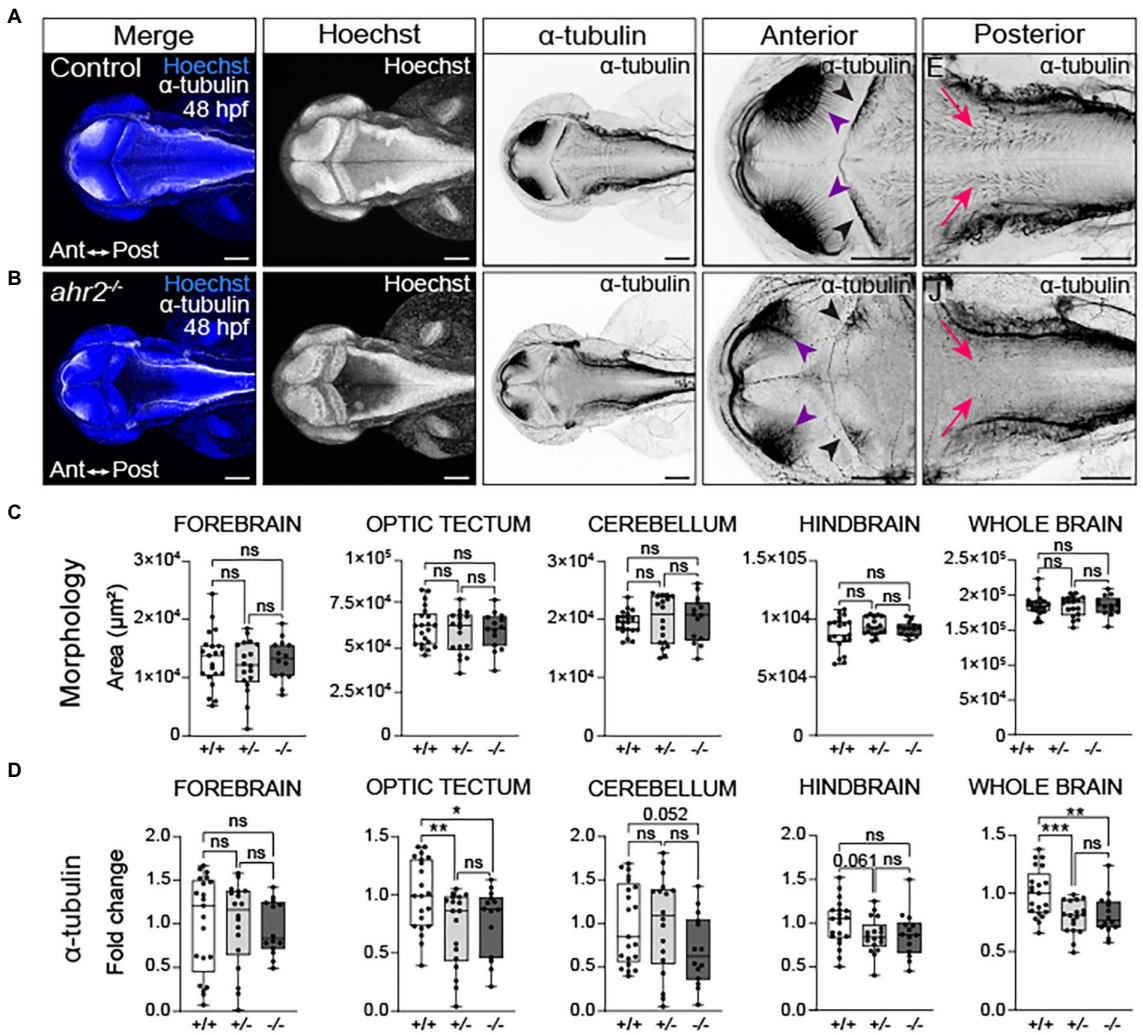
Loss of *ahr2* disrupts neural network formation without affecting brain size. Dorsal views of control **(A)** and homozygous *ahr2* mutant embryos **(B)** at 48 hpf stained with Hoechst (DNA, blue) to visualize the parenchyma and immunolabeled with an anti-acetylated α-tubulin antibody to mark axon tracts (white and inverted to black). **(C)** Area measurements of the forebrain, optic tectum, cerebellum, hindbrain, and whole brain at 48 hpf in wild-type *ahr2*, heterozygous *ahr2*, and homozygous *ahr2* mutant embryos. **(D)** Fold change of acetylated α-tubulin in the forebrain, optic tectum, cerebellum, hindbrain, and whole brain of heterozygous *ahr2*, and homozygous *ahr2* mutant embryos zebrafish relative to the mean acetylated α-tubulin area coverage of wild-type embryos. There were no apparent differences in the size of the forebrain, optic tectum, cerebellum, hindbrain, and whole brain in mutants when compared to controls **(C)**; however, acetylated α-tubulin coverage was significantly reduced in the optic tectum (purple arrowheads) and the whole brain of homozygous and heterozygous *ahr2* mutant embryos embryos relative to controls **(D)**. Acetylated α-tubulin coverage in the cerebellum (black arrowheads) and hindbrain (pink arrowheads) was also reduced, but this was not a statistically significant finding with the analysis pipeline used for quantification **(D)**. Statistical significance determined by Welch’s t-test, *p < 0.05, **p < 0.01, ***p < 0.001. *n* = 14–21 fish/group across 3 replicates. Anterior is left in all confocal micrographs. Scale bar = 100 μm. Magnification = 10x with varying zoom.

Similar to what was observed with toxicant-induced AHR gain-of-function, loss of one or both *ahr2* alleles disrupted axon tract development (Figure 8 and Supplementary Figure 6). Reductions in neural network formation were observed in optic tectum and whole brain in *ahr2* mutants relative to wild-type embryos (Figures 8A,B,D). Loss of one or both *ahr2* alleles disrupted development of the optic tectum neuropils with notable reductions of axon tracts projecting from the neuropils into the optic tectum at 48 hpf (purple arrowheads in Figures 8A,B). Loss of *ahr2* function also altered Purkinje cell morphology and axonal innervation of the cerebellum (black arrowheads in Figures 8A,B). However, unlike what was observed following TCDD exposure, loss of *ahr2* function did not alter whole brain morphology (Figure 7C). There were no observable differences in the area of the forebrain, optic tectum, cerebellum, hindbrain, or whole brain in *ahr2* null mutants.

## Discussion

### Identification of conserved and novel brain regions impacted By TCDD exposure

Human exposure to the potent AHR agonist TCDD increases the risk of developing neuropathologies, cancers, cardiovascular disease, diabetes, and neurodevelopmental disorders (Pocchiari et al., 1979; Assennato et al., 1989; Signorini et al., 2000; Kim et al., 2003; Pavuk et al., 2003; Mocarelli et al., 2008; Pesatori et al., 2009; Eskenazi et al., 2010; Warner et al., 2011; Nishijo et al., 2014; Pham et al., 2015; Tran et al., 2016). Previous research using vertebrate models has identified neurofunctional changes following TCDD exposure and identified the hippocampus, olfactory bulb, and cerebellum as targets of TCDD-induced neurotoxicity (Williamson et al., 2005; Collins et al., 2008; Sánchez-Martín et al., 2011; Latchney et al., 2013; Kimura et al., 2016; Hahn et al., 2017). In the present study, we used zebrafish to model the effects of TCDD exposure on embryonic brain development with the goal of identifying conserved and novel brain regions, developmental processes, and cell-types that are sensitive to AHR modulation.

Previous work suggested that toxicant induced AHR activation decreases whole brain volume in zebrafish independent of changes in body size (Hill et al., 2003, 2004). However, these studies assessed brain size at 168 hpf when edema, a marker of cardiac dysfunction, was present. Although the authors accounted for edema when quantifying brain size, the studies cannot distinguish between direct effects of TCDD exposure on neural development and secondary effects produced by changes in cardiac function, a known modulator of organogenesis (McQuillen et al., 2010; Cohen et al., 2016; Morton et al., 2017). We found TCDD exposure during embryogenesis altered brain development at 48 hpf, prior to changes in cardiac output. Specifically, we found that the size of the forebrain and optic tectum was reduced, which resulted in overall reduction in brain size at both 48 and 72 hpf. The change in area likely reflects a reduction in neurons within the measured brain regions, though we cannot rule a potential effect on the development of astrocytes, a plentiful cell type at 48 and 72 hpf. The observed regional reductions could reflect the disruption of a variety of neurodevelopmental processes with one potential mechanism being the disrupted development or differentiation of neural progenitor cells. Zebrafish have multiple waves of neurogenesis that precede the timepoints analyzed. A primary wave of neurogenesis occurs prior to 48 hpf, which establishes a brain scaffold and is followed by a secondary post-embryonic wave that substantially builds on the scaffold between days 48 and 96 hpf (Korzh et al., 1993; Chapouton and Bally-Cuif, 2004). New neurons continue to be added to existing regions as larvae mature and exhibit more complex behaviors, such as foraging and predatory evasion, which are particularly associated with neurogenesis in optic tectum (Marachlian et al., 2018; Boulanger-Weill and Sumbre, 2019). Altered AHR signaling has previously been shown to adversely affect the development of different progenitor populations including neural progenitor cells in the mammalian hippocampus, granule neuron precursor cells in the mammalian cerebellum, and proepicardial progenitor cells during zebrafish cardiogenesis (Collins et al., 2008; Latchney et al., 2013; Plavicki et al., 2013; Bock, 2017). In addition, we report in this manuscript that TCDD exposure disrupts the development of oligodendrocyte precursor cells and their derivatives. However, the observed reduction in this cellular population alone is not sufficient to explain the reduction in overall brain size at 48 hpf.

The observed changes in brain size could also reflect an increase in cell death or an inhibition of cell cycle. Dong et al. (2001) demonstrated an increase in apoptosis in the dorsal midbrain following a 24-h exposure to TCDD at a concentration of 1 part per billion (Dong et al., 2001). While the increase was statistically significant, the overall extent of apoptosis with this substantially longer exposure paradigm does not appear sufficient to produce the changes in brain size observed in our studies. However, this does not exclude changes in cell-cycle as a potential factor contributing to the observed changes in brain size. The impact of AHR agonist exposure on cell cycle appears to be tissue and stage specific with some studies indicating an increase in proliferation following exposure and others demonstrating an impairment (increase: (Andrysík et al., 2007; Umannová et al., 2007; Xu et al., 2014; Gao et al., 2016; Wu et al., 2019) decrease: (Jönsson et al., 2007; Faust et al., 2013; Li et al., 2014; Burns et al., 2015). Future studies should address the impact of TCDD exposure on cell cycle during early embryonic waves of neuro- and gliogenesis.

Prior work examining how TCDD exposure affects zebrafish brain size examined gross brain morphology, but did not identify which brain regions or nuclei were specifically affected by exposure. Here, we provided further insight into which brain regions are adversely affected by TCDD exposure, identifying regions previously shown to be sensitive to AHR modulation in mammals as well as new areas of interest. Previous research in mammals demonstrated that aberrant AHR signaling disrupts cerebellar development. Studies in rodents found that cerebellar granule cell precursors, which give rise to the excitatory neurons of the cerebellum, are a target of developmental TCDD-induced neurotoxicity (Williamson et al., 2005; Collins et al., 2008; Sánchez-Martín et al., 2011). Dever et al. (2016) demonstrated that cell-specific *Ahr* deletion in cerebellar granule cell precursors impairs proliferation and leads to a reduction in cell number. Consistent with what has been observed in mammals, we found zebrafish cerebellum was sensitive to both gain and loss of AHR function. TCDD-induced AHR activation, neuron-specific expression of caAHR, and loss of *ahr2* altered connectivity within the cerebellum; however, only TCDD exposure affected gross cerebellar morphology. Taken together, these results identify the cerebellum as a conserved target of TCDD toxicity and sensitive to AHR modulation. The conservation of the cerebellar phenotype across vertebrate species suggests that zebrafish studies of AHR-induced toxicity are relevant to mammals and supports the use of the zebrafish model for identifying new cellular and regional targets that can be subsequently examined in mammalian models.

We also identified new brain regions of interest that are affected by TCDD and report that TCDD exposure disrupted development of the optic tectum and the forebrain, which contains the olfactory bulbs, the habenular nuclei, and will ultimately give rise to the amygdaloid complex (Mueller, 2022). The affected brain regions are essential for sensory processing and coordinating responses to stressful and rewarding stimuli. Altered sensory processing is associated with human developmental neuropathologies; therefore, it would be useful for future developmental neurotoxicology studies to assess the impact of complex, environmentally relevant mixtures on sensory system development in zebrafish. From an ecological perspective, the observed disruptions could also impair the fitness of wild fish species.

### Modulation of AHR signaling alters neural connectivity

The lasting impact of aberrant connectivity on brain function underscores the importance of studying how neural network formation is affected by contaminant exposures. Previous research identified AHR agonist-induced changes in the PNS of Atlantic seabream as well as structural differences and functional changes in larval zebrafish (Ton et al., 2006; Iida et al., 2013; Knecht et al., 2017; Holloway et al., 2021). Ton et al. (2006) found that TCDD exposure qualitatively reduced axon tracts in the developing zebrafish brain. Our findings significantly build on these initial reports and use high-resolution confocal z-series in multiple orientations to identify specific brain nuclei, commissures, and tracts affected by TCDD exposure. Furthermore, we developed an analysis pipeline to quantify changes in acetylated α-tubulin coverage across brain regions and found early embryonic TCDD exposure significantly reduced neural network formation in the forebrain, optic tectum, cerebellum, and the whole brain. Given the regional changes in brain size, it is also possible that reduced numbers of neurons altered the patterns of connectivity in the developing brain. Concurrent with the observed disruptions in axon tract development, TCDD exposure decreased synaptic vesicle immunolabeling at 48 hpf. The structural and connectivity changes observed following TCDD exposure could also occur following exposure to other AHR agonists, which would provide a structural correlate to the behavioral phenotypes reported following benzo[a]pyrene exposure. Consistent with our observation that connectivity is disrupted in zebrafish, studies using multiple model systems have found that modulating AHR signaling alters dendritic development. Studies using the mouse model found perinatal TCDD exposure causes abnormal dendritic growth in the hippocampus (Kimura et al., 2015) and, in cultured rat cortical cells, TCDD exposure decreased dendrite complexity (Cho et al., 2002). Furthermore, studies in *Drosophila* demonstrated that the overexpression and the loss of the AHR homolog *spineless* alters dendritic branching with overexpression reducing dendritic branching and genetic loss increasing branching (Kim et al., 2006). The consistent phenotypes observed across model organisms demonstrates the importance of proper AHR activity during neural networks formation and highlights axonal tract and dendrite development as important targets of TCDD-induced neurotoxicity.

Given the role of the *Drosophila* homolog of AHR, *spineless*, in eye development, the observed changes in the size and organization of the optic tectum could reflect disrupted development of the retina and the corresponding loss and/or impaired targeting of retinal-tectal projections (Wernet et al., 2006; Bianco et al., 2011). Alternatively, given that axon pathfinding defects were observed in multiple brain regions, the aberrant AHR activation could dysregulate key components of signaling pathways that are essential for axon guidance in many brain regions or impair fundamental components of axon outgrowth such as calcium dynamics or microtubule assembly. Previous work has shown prenatal exposure to TCDD induces expression of the *Sema3b* and *Sema3g* in the olfactory bulb, postnatal mouse brain at postnatal day 3 (Kimura et al., 2016). Additional work is necessary to fully define the olfactory bulb phenotypes in TCDD-exposed larvae and to determine if semaphorin signaling is altered following TCDD exposure in zebrafish.

The effects of *ahr2* loss and TCDD exposure on neural network formation suggest neurons are cellular targets and susceptible to modulations in AHR activity. Axonal innervation in the cerebellum was significantly reduced when a caAHR construct was specifically expressed in differentiated neurons. However, expression of a caAHR in differentiated neurons was not sufficient to disrupt axon tract development in the forebrain, optic tectum, hindbrain, or whole brain. A number of factors are important to consider when interpreting the phenotypic differences observed between TCDD-induced AHR activation and neuron-specific caAHR expression. Previous reports have shown that different AHR agonists can induce different transcriptional profiles (Goodale et al., 2015). The caAHR construct used in our studies is a zebrafish-mouse chimera (Lanham et al., 2014) and the chimeric AHR and endogenous *ahr2* could produce different transcriptional profiles that result in phenotypic differences. It is also important to note that there is an inherent lag in the Gal4/UAS system and, in order for the caAHR construct to be expressed, both Gal4 and UAS must first be transcribed. Therefore, the caAHR construct may not be present when the axon growth cone is first formed and responding to directional signals. It is also possible that toxicant-induced AHR activation affects the cells that secrete guidance cues during early network formation.

### Oligodendrocytes are targets of developmental TCDD toxicity

Impaired oligodendrocyte development is associated with multiple neurodegenerative disorders including cerebral palsy, spinal cord injury, stroke, and multiple sclerosis (Káradóttir and Attwell, 2007; Kang et al., 2013). A number of lines of evidence indicate that altered AHR signaling may disrupt glial development. Pathway analyses in mice suggests myelin gene dysregulation post gestational TCDD exposure (Gileadi et al., 2021). Additional developmental studies in mice focused on gene expression data proposed that *in utero* TCDD exposures may have long-term effects on myelin and gliogenesis of the adult CNS (Fernández et al., 2010). Furthermore, adult TCDD exposure in rats caused inflammation, neuronal vacuolization, and demyelination in the hippocampus (Rosińczuk et al., 2015). These studies place emphasis on the terminal function of oligodendrocytes, i.e., myelination; however, they have not investigated the effects TCDD exposure on oligodendrocyte precursor cells. Using *in vivo* confocal microscopy, we show that embryonic TCDD exposure disrupts oligodendrocyte precursor development with significant reductions in total number of oligodendrocyte precursor cells in the zebrafish hindbrain at 48 and 72 hpf. Given the observed impairment of oligodendrocyte development, another outstanding question that warrants future attention is whether TCDD exposure affects multiple glial subtypes and, if so, if glia continue to be sensitive to exogenous AHR activation at later stages of development and in the mature brain.

The observed loss of oligodendrocyte precursor cells in TCDD exposed embryos and larvae may be due to the altered or reduced expression of the SoxE genes. The SoxE genes, a family of transcription factors that includes *Sox9*, *Sox10*, and *Sox8*, are important for glial cell fate determination and differentiation. Loss of *Sox9* in mice has been shown to reduce oligodendrocyte cell counts and increases neuronal subtypes in the spinal cord, suggesting that *Sox9* alters the developmental trajectory of CNS stem cells and promotes gliogenesis (Finzsch et al., 2008). Concurrent loss of *Sox9* and *Sox10* in oligodendrocyte precursor cells in mice altered the number and migration patterns of precursors, reducing the overall quantity of differentiated oligodendrocytes (Finzsch et al., 2008). Loss of function studies of *sox9a* and *sox9b* found that both orthologs of mammalian *Sox9* were involved in the specification of oligodendrocyte precursor cells in the zebrafish hindbrain (Esain et al., 2010). The interpretation of these studies is complicated by the finding that the mutants used for analysis were large deletion mutants that affected many genes and not just the *Sox9* orthologs. The phenotypes are, however, consistent across species, lending support for *sox9a* and *sox9b* being regulators of gliogenesis. In zebrafish, TCDD exposure results in the downregulation of *sox9b* in the heart, jaw, and regenerating fin (Andreasen et al., 2006; Xiong et al., 2008; Hofsteen et al., 2013). The observed impairment in gliogenesis in TCDD exposed embryos may result from the downregulation of *sox9b* or the down regulation of multiple SoxE genes. Future experiments are necessary to address whether additional SoxE genes are, indeed, targets of the AHR signaling pathway.

## Conclusion

Our studies demonstrate that proper modulation of AHR signaling pathway is required for embryonic brain development. We identified both conserved and novel brain regions that are targets of TCDD induced neurotoxicity and provide a foundation for future studies to determine the molecular mechanisms by which aberrant modulation of the AHR signaling pathways disrupts critical steps in early brain development. Together, our data demonstrate that zebrafish are a sensitive and relevant model for understanding the impact of environmental contamination on brain health.

## Data availability statement

The original contributions presented in the study are included in the article/Supplementary material, further inquiries can be directed to the corresponding author. Data will be freely shared upon request.

## Ethics statement

The animal study was reviewed and approved by Brown University Institutional Animal Care and Use Committee.

## Author contributions

NM and JP designed the experiments and figures as well wrote the manuscript. MY, JG, and LE generated critical preliminary data. MY generated the *Tg(UAS:caAHR:2A-tRFP:cryaa:EGFP)* line. NM, RP, MK, and JP collected the primary data sets for the initial manuscript submission. NM, RP, FN, LT, LC-R, and JP performed data analysis. LT, MC, LC-R, MK, and JP generated data, text, and figures for the revised manuscript. JP secured funding. All authors contributed to the article and approved the submitted version.

## Funding

This work was funded by the NIEHS Training in Environmental Pathology T32 (ES007272), which provided support to NM and MK. MK was also supported by an NIEHS F32 (ES032650), JP was supported by an NIEHS K99/R00 (ES023848), a CPVB Phase II COBRE (2PG20GM103652), and an NIEHS ONES award (ES030109).

## Conflict of interest

The authors declare that the research was conducted in the absence of any commercial or financial relationships that could be construed as a potential conflict of interest.

## Publisher’s note

All claims expressed in this article are solely those of the authors and do not necessarily represent those of their affiliated organizations, or those of the publisher, the editors and the reviewers. Any product that may be evaluated in this article, or claim that may be made by its manufacturer, is not guaranteed or endorsed by the publisher.

## Abbreviations

TCDD, dioxin: 2,3,7,8-tetrachlorodibenzo-[p]-dioxin
AHR: Aryl hydrocarbon receptor
caAHR: Constitutively active AHR
SV2: Synaptic vesicle glycoprotein 2
hpf: hours post-fertilization
UAS: Upstream activating sequence
CNS: Central nervous system
PNS: Peripheral nervous system
