# Supplementary figures and images for: Proper modulation of AHR signaling is necessary for establishing neural connectivity and oligodendrocyte precursor cell development in the embryonic zebrafish brain
Source: Front Mol Neurosci. 2022 Nov 29;15:1032302. doi: 10.3389/fnmol.2022.1032302 (PMC9745199; doi:10.3389/fnmol.2022.1032302)

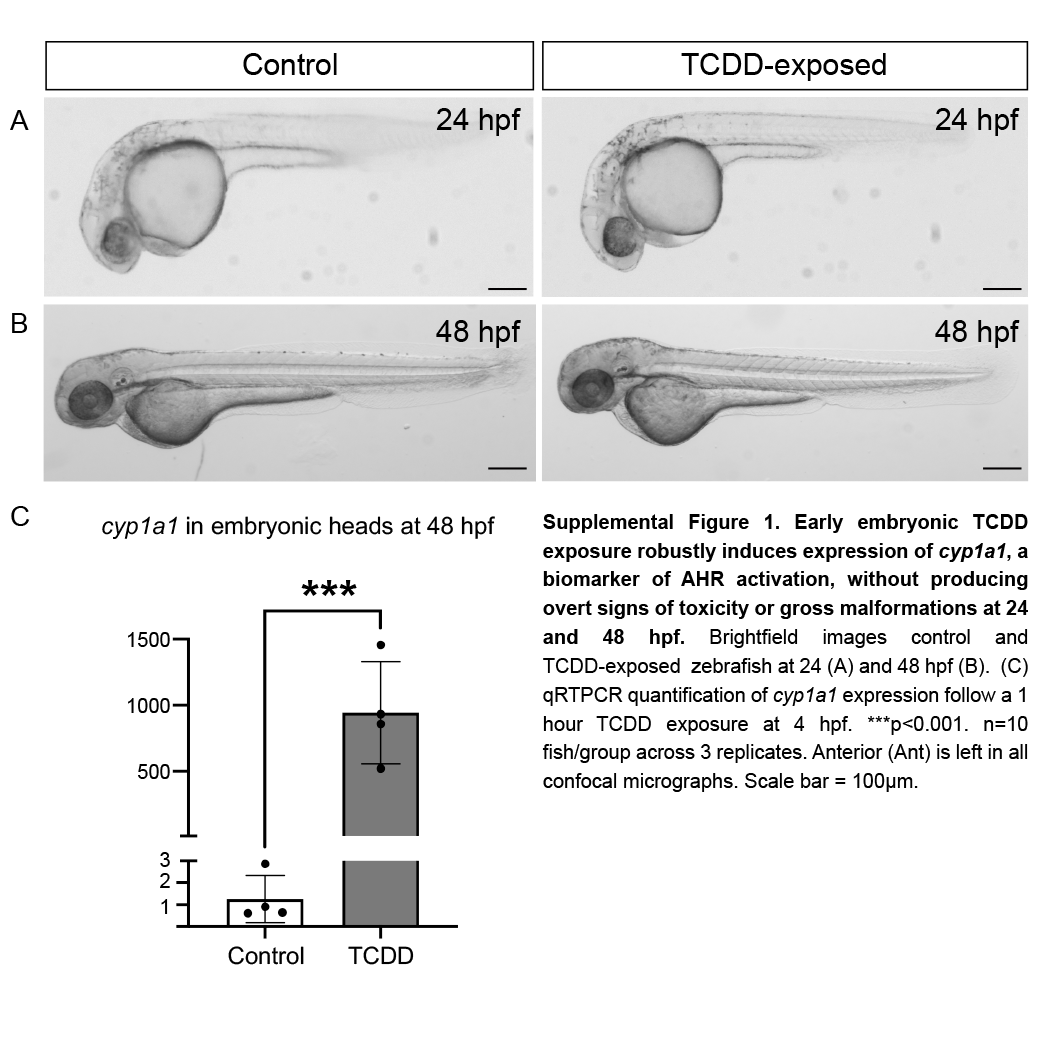

Supplement: Supplementary file 1 [file Image_1.TIF]

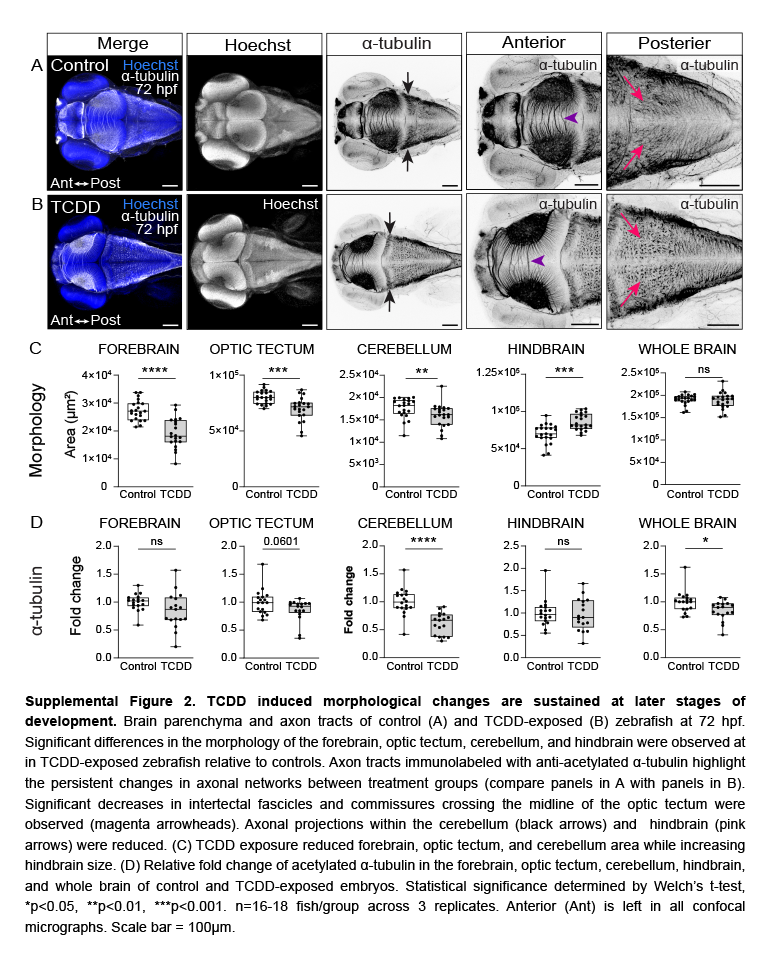

Supplement: Supplementary file 2 [file Image_2.TIF]

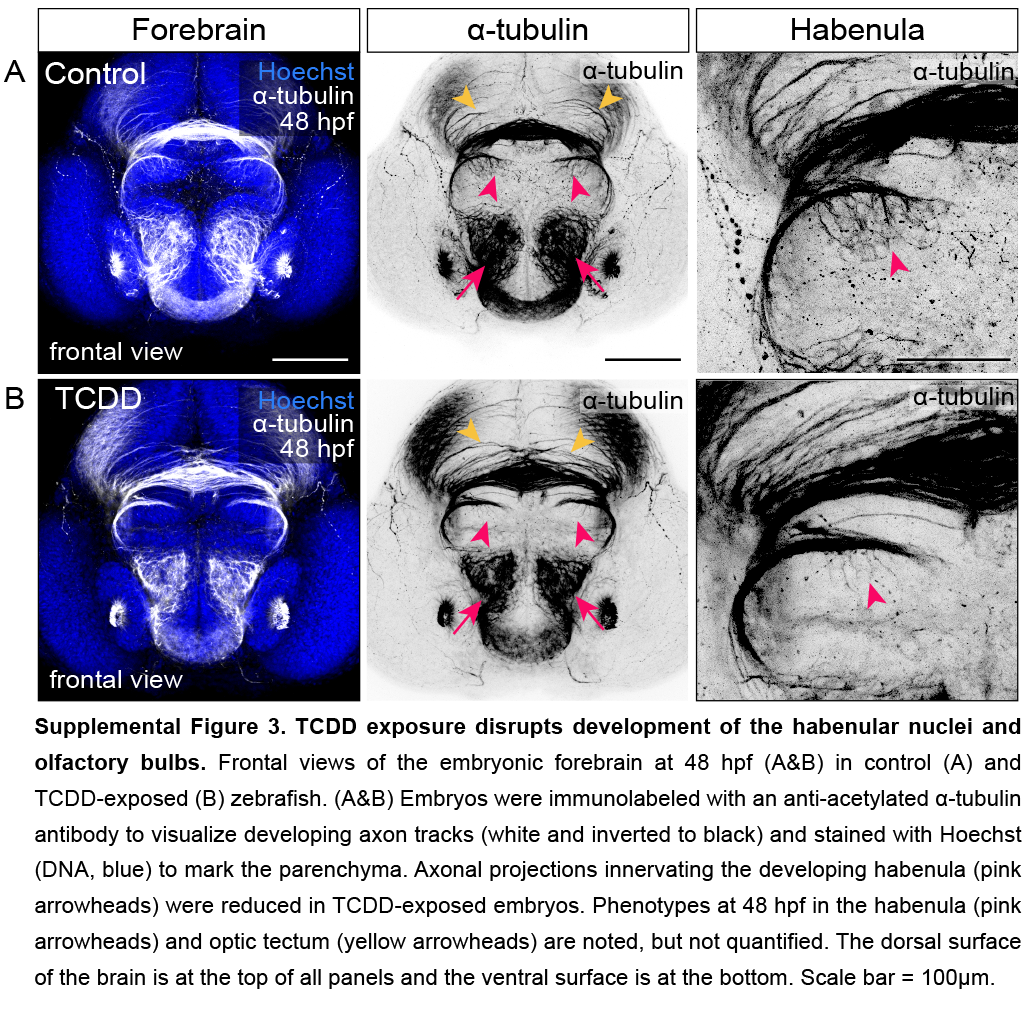

Supplement: Supplementary file 3 [file Image_3.TIF]

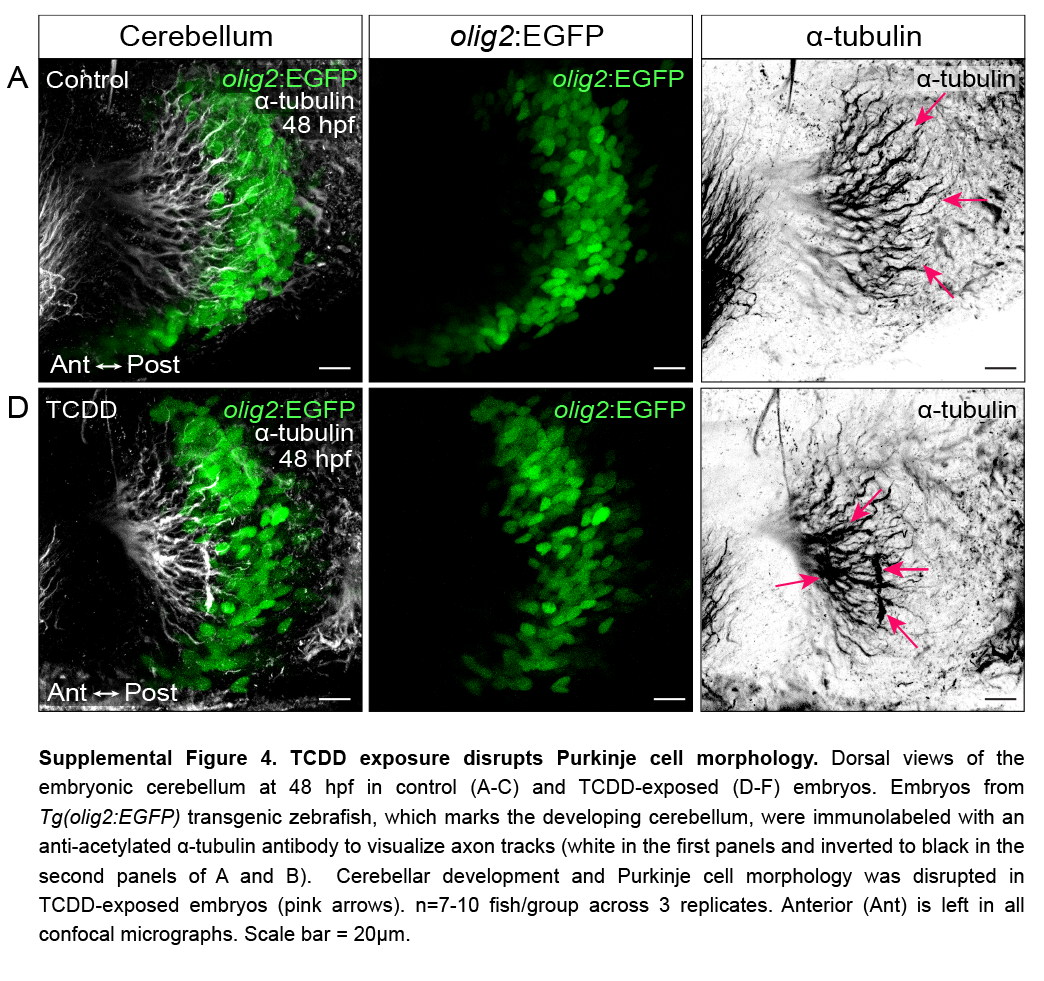

Supplement: Supplementary file 4 [file Image_4.TIF]

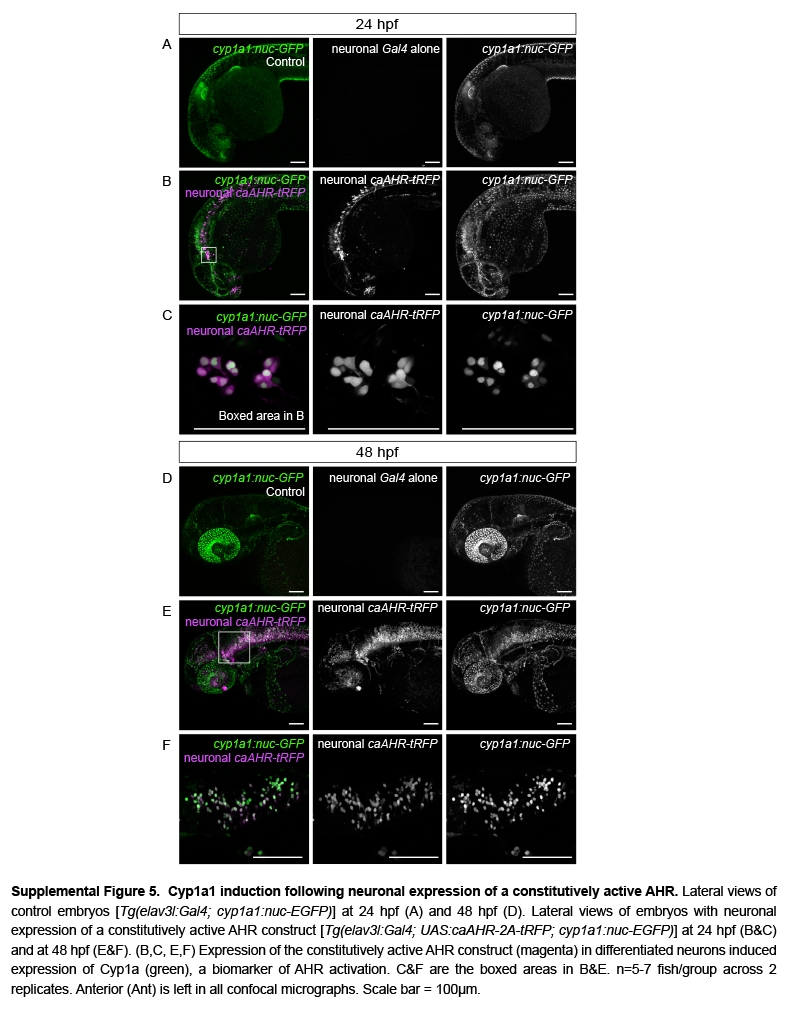

Supplement: Supplementary file 5 [file Image_5.TIF]

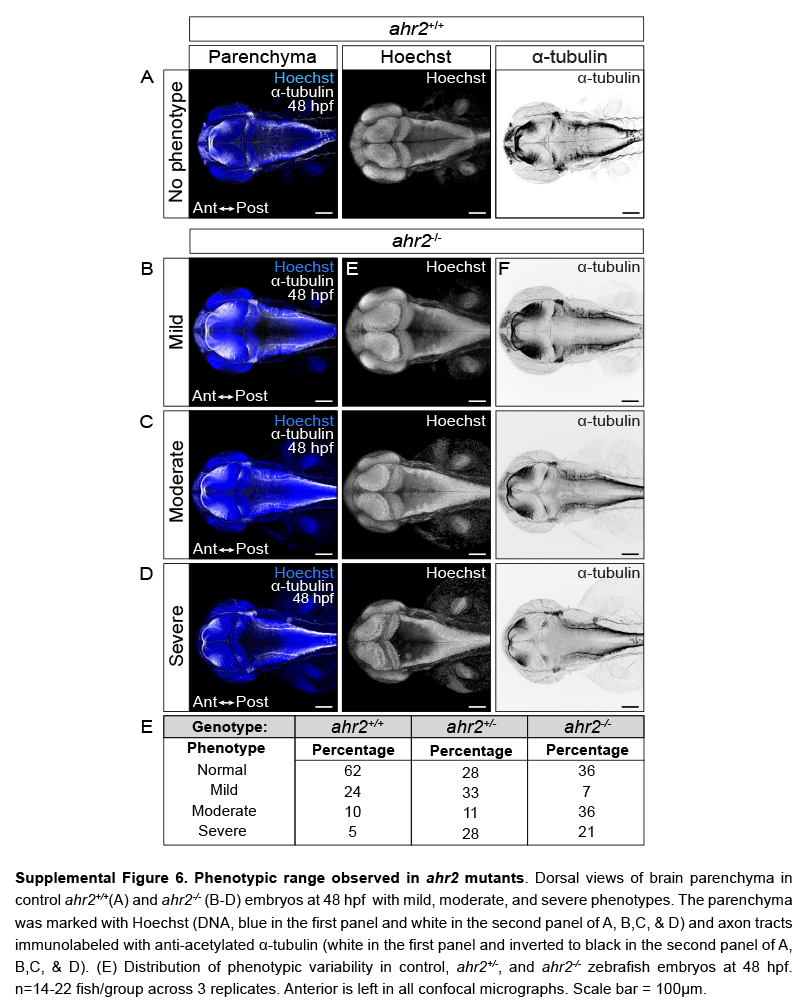

Supplement: Supplementary file 6 [file Image_6.TIF]
