# Supplementary material for: Proper modulation of AHR signaling is necessary for establishing neural connectivity and oligodendrocyte precursor cell development in the embryonic zebrafish brain
Source: Front Mol Neurosci. 2022 Nov 29;15:1032302. doi: 10.3389/fnmol.2022.1032302 (PMC9745199; doi:10.3389/fnmol.2022.1032302)
Supplement: Supplementary file 7 [file Table_1.DOCX]

**Table 1. Transgenic *Danio Rerio* lines used in manuscript**

| Transgenic Line | Tissue/Function | Reference |
| --- | --- | --- |
| *Tg(olig2:EGFP)^vu12^* | OPCs and motor neurons | Shin et al., 2003 |
| *Tg(sox10:RFP)* | Neural crest and differentiated oligodendrocytes | Kucenas et al., 2008 |
| *Tg(elavl3:Gal4-VP16)* | Elavl3 promoter fused Gal4-driver enabling neuronal specific expression of genes fused to an upstream-activating sequence (UAS) | Kimura et al., 2008 |
| *Tg(UAS:caAHR:2A-tRFP:cryaa:EGFP)* | Paired with Gal4-driver, enables expression of constitutively active AHR in cell-specific manner | Current article |
| *ahr2^uab147^* | Frameshift mutation in AHR2, resulting in loss-of-function and sensitivity to TCDD toxicity | Souder and Gorelick, 2019 |
